# The role of intestinal microbiota in murine models of acetaminophen-induced hepatotoxicity

**DOI:** 10.1111/liv.12689

**Published:** 2014-10-08

**Authors:** Lucia A Possamai, Mark JW McPhail, Wafa Khamri, Bishan Wu, Danilo Concas, Mark Harrison, Roger Williams, Roger D Cox, I Jane Cox, Quentin M Anstee, Mark R Thursz

**Affiliations:** 1 Department of Hepatology, Imperial College London W2 1NY, UK; 2 MRC Mammalian Genetics Unit, Harwell, Oxford, OX11 0RD, UK; 3 Institute of Hepatology, Foundation for Liver Research, 69-75 Chenies Mews, London WC1E 6HX, UK; 4 Institute of Cellular Medicine, Newcastle University, Newcastle upon Tyne, NE2 4HH, UK

**Keywords:** Acetaminophen, acute liver failure, germ-free, microbiota, metabonomics

## Abstract

**Background & Aim:**

Variations in intestinal microbiota may influence acetaminophen metabolism. This study aimed to determine whether intestinal microbiota are a source of differential susceptibility to acetaminophen-induced hepatotoxicity.

**Methods:**

Conventionally housed C3H/HeH (CH) and C3H/HeH germ free (GF) mice were administered a 200mg/kg IP dose of acetaminophen. The severity of hepatotoxicity at 8 hours was assessed by histology and biochemical indices. A urinary metabolic profile was obtained using ^1^H-NMR. Baseline hepatic glutathione content and CYP2E1 expression were quantified. An additional group of C3H/HeJ (LPS-r) mice were assessed to determine the contribution of LPS/TLR4 signalling.

**Results:**

Baseline glutathione levels were significantly reduced (p=0.03) in GF mice. CYP2E1 mRNA expression and protein levels were not altered. Inter-individual variability did not differ between GF and CH groups. No significant differences in the extent of hepatocellular injury (ALT or percentage necrosis) were demonstrated. However, a milder acute liver failure (ALF) phenotype was shown in GF compared with CH mice, with reduced plasma bilirubin and creatinine and increased blood glucose. Differential acetaminophen metabolism was demonstrated. GF mice displayed a higher urinary acetaminophen-sulphate:glucuronide ratio compared with CH (p=0.01). Urinary analysis showed metabolic differentiation of GF and CH groups at baseline and 8 hours(cross-validated ANOVA p=1x10^-22^). Interruption of TLR4 signalling in LPS-r mice had additional protective effects.

**Conclusion:**

Variations in intestinal microbiota do not fully explain differential susceptibility to acetaminophen-induced hepatotoxicity. GF mice experienced some protection from secondary complications following acetaminophen overdose and this may be mediated through reduced TLR4/LPS signalling.

## Introduction

Acetaminophen is a commonly used analgesic and anti-pyretic medicine. Since it was first recognised as a potent hepatotoxin in the 1960s, acetaminophen overdose has become the most common cause of acute liver failure in the UK and USA (1–3).

In patient populations there is a wide range of inter-individual susceptibility to the hepatotoxic effects of acetaminophen, which is incompletely explained by known risk factors such as chronic alcohol abuse or nutritional deficiencies(4). Patient outcome following acetaminophen-induced acute liver failure does not correlate with the dose of acetaminophen ingested (5). Recently, evidence has emerged that a proportion of the adult population experience elevation in liver transaminase levels indicative of hepatocellular damage following the consumption of doses of acetaminophen within the therapeutic range (4g/24hrs) over short periods (7-14 days) (6, 7).

Murine models of acetaminophen toxicity are commonly used to examine the underlying mechanisms of hepatocellular damage in this condition and more generally as a model of acute, sterile liver inflammation. Unexplained variability in the response of genetically identical mice to toxic doses of acetaminophen is widely recognised and it has been hypothesised that this variability may be accounted for by differences in intestinal microbiota (8).

Differences in host intestinal microbiota are increasingly recognised as a potential source of inter-individual variation in response to drugs and toxins (9). In the particular case of acetaminophen handling, Clayton et al found an association between pre-dose, gut-derived urinary metabolites and response to a therapeutic dose of acetaminophen in human subjects. Higher levels of pre-dose urinary p-cresol sulphate predicted a reduction in the acetaminophen-sulphate: acetaminophen-glucuronide ratio in post-dose urine (10). It was postulated that gut-derived microbial metabolites might increase acetaminophen toxicity.

The theoretical mechanisms by which gut microbiota might influence host susceptibility to acetaminophen-induced hepatotoxicity are numerous. Firstly it has been shown that bacterial-derived metabolites are capable of up-regulating some cytochrome p450 hepatic enzymes (11, 12). This has been demonstrated for members of the CYP family including CYP3A4, CYP1A2, CYP2A6, though not for CYP2E1 itself. Secondly, depletion of hepatic sulphonation capacity through competitive inhibition by bacterially-derived metabolites can occur and could theoretically drive a greater proportion of the drug to be oxidised to toxic product by the CYP450 enzyme system. Thirdly, intestinal bacteria have been shown to hydrolyse conjugated acetaminophen to release free drug during the enterohepatic circulation of acetaminophen metabolites, thus potentially enhancing an individual’s exposure to the active drug for any given dose ingested (13, 14). Finally, bacterially-derived products, such as LPS, trafficking in the portal vein to the liver have been implicated in the exacerbation of liver disorders through their activation of hepatic TLR receptors. In acetaminophen-induced hepatotoxicity, the blocking of endogenous LPS activity with an inhibitory peptide has been shown to ameliorate liver injury, suggesting a role for endogenous microbial products in the pathophysiology of drug-induced hepatic injury (15).

In the present study we hypothesised that intestinal microbiota contribute to the development and severity of acute acetaminophen-induced hepatotoxicity and that this effect is partly modulated through LPS-TLR4 signalling. The influence of intestinal microbiota on susceptibility to hepatotoxicity was assessed in germ-free and conventionally housed mice. Baseline differences in factors known to influence hepatotoxicity, such as hepatic glutathione reserves and CYP2E1 expression were first examined and then the severity of hepatotoxicity assessed with biochemical indices and histological measurement of necrosis. A third group of LPS-resistant mice were used to determine whether intact LPS-TLR signalling was an important determinant of susceptibility to acetaminophen-induced hepatotoxicity. The influence of gut microbiota on the metabolism of acetaminophen was additionally studied in germ-free and conventionally housed mice using urinary NMR profiling.

## Materials and Methods

### Animals

All research using live animals was approved by the local ethics committee (MRC Harwell) and carried out under Home Office supervision in accordance with the Animal (Scientific Procedures) Act 1986 (UK). All efforts were taken to minimize animal suffering. Three experimental groups of male animals aged 8-11 weeks were used*.* The control group (CH) was composed of 20 C3H/HeH mice housed under standard ‘specific pathogen free’ conditions (12 hour light/dark cycle, temperature 21 ± 2°C, humidity 55 ± 10%) and provided with a standard commercial diet (SDS, UK) and *ad libitum* access to drinking water. The group (GF) of 9 C3H/HeH mice was from a germ-free colony maintained at the same facility under strict sterile conditions in a positive pressure isolator (Harlan Isotec). Complete sterility of animals within the germ free colony was validated by regular swabs, faeces and urine cultures and bacterial 16sRNA PCR assays. The third group (LPS-r) was composed of 10 C3H/HeJ mice maintained under SPF conditions as per control group. C3H/HeJ mice are derived from the same founder strain as C3H/HeH mice and share significant genetic homology, but are known to carry a missense mutation within the third exon of their TLR4 receptor gene (predicted to replace proline with histidine at position 712) that renders them relatively LPS-resistant (16).

A further 10 GF and 10 CH mice between 8-12 weeks of age were culled without acetaminophen dosing. Liver tissue was snap frozen in liquid nitrogen then stored at -80°C.

### Acetaminophen dosing

Following overnight fast, mice were weighed and a single intraperitoneal dose of 200mg/kg of acetaminophen dissolved in warm saline was administered.

### Sample collection for biochemistry and histology

Blood was collected via cardiac puncture at eight hours (T8), under terminal anaesthesia with pentobarbital. The liver was fixed in formalin.

Biochemical analysis of plasma T8 samples was performed on a Beckman Coulter AU680 semi-automated clinical chemistry analyser, using the manufacturer’s instructions, parameter settings and reagents for measurement of Alanine Transaminase (ALT), Bilirubin, Glucose and Creatinine.

Formalin-fixed liver tissue was embedded in paraffin, sectioned and stained with haematoxylin and eosin (H&E). A quantitative necrosis score was generated by a point-scoring technique using a digital 100 square grid over each of the 10 images per mouse with the reviewer blinded to the experimental group.

### Gluathione Assay

Baseline liver samples from 10 GF and 10 CH mice were assayed for total gluathione using the ApoGSH™ Glutathione Detection Kit (BioVision, Milpitas, CA) according to manufacturers instructions.

### CYP2E1 Expression by RT-PCR and ELISA

RNA was extracted from snap frozen liver tissue using TRIzol® reagent (Life Technologies, Carlsbad, CA) according to manufacturers instructions. 1μg of RNA was reverse transcribed using a Quantitect RT Kit (Qiagen, Dusseldorf, Germany). Gene Expression Assay Mm00491127 specific to murine CYP2E1 was used and B2M, SDHA and 18s ribosomal RNA served as reference genes (TaqMan® Gene Expression Assays Mm00437762, Mm01352363 and Mm03928990 respectively) (Life Technologies, Carlsbad, CA). Biological replicates of 5 GF and 5 CH mice were used and each reaction run in triplicate. Results were analysed using a ΔΔC_T_ method using the geometric means of the reference genes.

Snap frozen liver tissue was thoroughly homogenised, protein content measured and normalised to 1mg/ml. CYP2E1 levels in the liver homogenates were measured by ELISA (CSB-EL006425MO, Cusabio, Wuhan, China) according to the manufacturers instructions.

### Hepatic cytokine measurements

Hepatic cytokines (TNFα, IL-1β, IFNγ and IL-6) were measured in liver homogenates (as above). 10ug of total protein was used per sample and cytokines quantified in duplicate using a MSD^®^ Multi-spot cytokine assay (Mesoscale Discovery^®^ Rockville, MD) according to the manufacturers instructions.

### Urinary NMR metabolic profiling

Urine samples were collected from all mice 24 hours prior to acetaminophen dosing (T-24 unfasted) and at baseline (T0) from fasted mice. Urine was then collected at two hours post-dosing from the germ-free mice and 10 of the CH controls for quantification of acetaminophen metabolites (T2). Finally urine samples were collected from all GF and CH mice at eight hours post-dosing (T8).

All urine samples were collected by clean catch into a sterile microcentrifuge tube that was placed at 4°C for a maximum of 1 hour before storage at -80°C.

In preparation for NMR studies, a urine volume of 10μl was mixed with 20μl sterile water and 30μl of 0.2M phosphate buffer (pH7.4, containing 20% D_2_O to provide an NMR field frequency lock and 0.5mM Trimethylsilyl-propionic acid (TSP) for internal chemical shift reference). The solution was transferred to a 1.7mm OD capillary tube, placed within a 5mm micro NMR tube (New Era, Vineland, New Jersey, USA). Urinary NMR *s*pectra were acquired using a JEOL 500 MHz Eclipse+ NMR spectrometer housed at MRC Harwell. Water presaturation was used for all data acquisitions. The spectral width was 15ppm, pulse angle 90°, acquisition time 4.36s and relaxation delay 2s. 32K data points were acquired per collect and 256 transients were summated. The receiver gain was constant for all samples. The resulting free induction decay was zero filled and multiplied by an exponential function corresponding to 0.3Hz line broadening prior to Fourier Transformation. The NMR spectra were manually phased using the JEOL Delta.

### Data Analysis

Prior to statistical analysis all NMR spectra were baseline corrected to a 4^th^ degree polynomial, zero filled by a factor of 2 and referenced with the TSP peak set to 0.00ppm using KIA version 8.x (Bio-Rad, Philadelphia, USA). NMR spectral resonances were assigned according to the literature (17). The resonances attributable to residual water and urea (δ 4.6-6.4ppm) were excluded from further analysis. NMR spectra were normalised to the total spectral integral in the range δ = 0.2-10 ppm (excluding 4.6-6.4ppm). Spectra were bucketed (total buckets 937) using the Intelligent Bucketing algorithm and mean centred. Principal Components Analysis (PCA) was used as an un-supervised method for data visualisation and outlier identification. Supervised regression modelling was performed on the mean centred data set using Partial Least Squares Discriminant Analysis (PLS-DA) within Pirouette v 4.0 (Infometrix, Bothell, WA). Validation was performed using leave one out cross validation. Individual metabolites were integrated within KnowItAll, summed and normalised as above and compared using the Kruskall-Wallis test within GraphPad Prism version 4 (SanDiego, CA).

Plasma biochemistry results were compared with a Kruskall Wallis test and post-hoc Dunn’s testing to account for multiple comparisons.

## Results

### Baseline hepatic glutathione and CYP2E1 levels

**Figure. 1:**
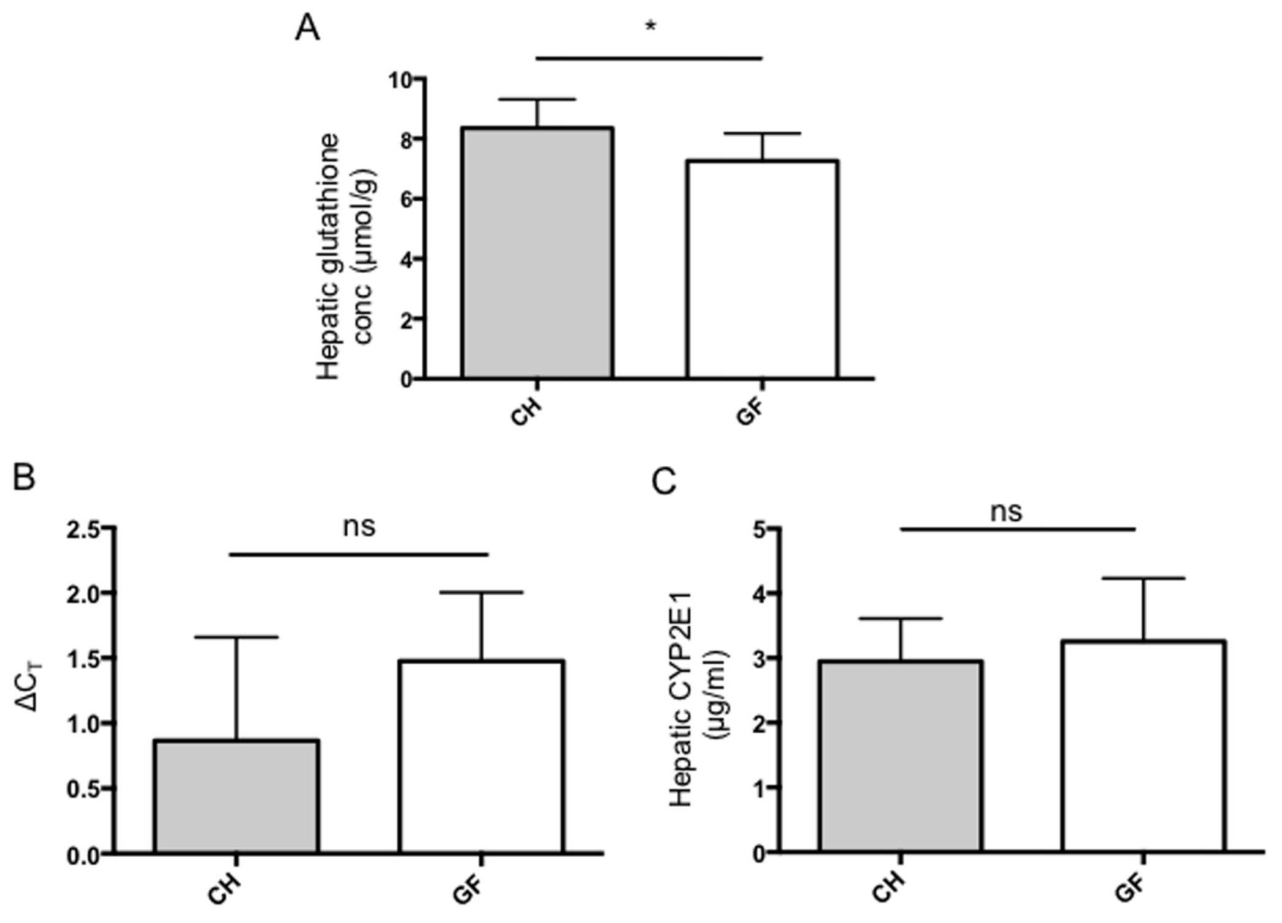
(A) Baseline hepatic glutathione levels in acetaminophen-naïve mice showing reduced glutathione stores in GF mice (P=0.028, n=10 in each group) (B) Hepatic CYP2E1 expression in acetaminophen-naïve GF and CH mice as determined by RT-PCR. Graph showing ΔCT values calculated by comparison of CYP2E1 expression with geometric mean of 3 reference genes (B2M, R18s, SDHA) (p=0.19, n=5 in each group) (C) Hepatic CYP2E1 levels in acetaminophen-naïve GF and CH mice determined by ELISA.

Total hepatic glutathione levels were quantified in GF and CH acetaminophen-naïve mice to determine steady state glutathione stores. Germ free mice showed a significantly reduced hepatic glutathione content (mean 7.26 +/- 0.92 μmol/g of liver tissue) compared with CH mice (mean 8.36 +/- 0.95 μmol/g of liver tissue) (p=0.029 Mann Whitney U test, figure 1A).

The relative hepatic expression of CYP2E1, which is the predominant enzyme responsible for metabolising acetaminophen to its reactive metabolite NAPQI, was determined in the two groups using RT-PCR and levels of protein quantified by ELISA. There were no significant differences in gene expression or protein levels of CYP2E1 between GF and CH mice (figure 1B and 1C respectively).

### Post-dosing biochemistry, histology & cytokine measurements

**Figure 2:**
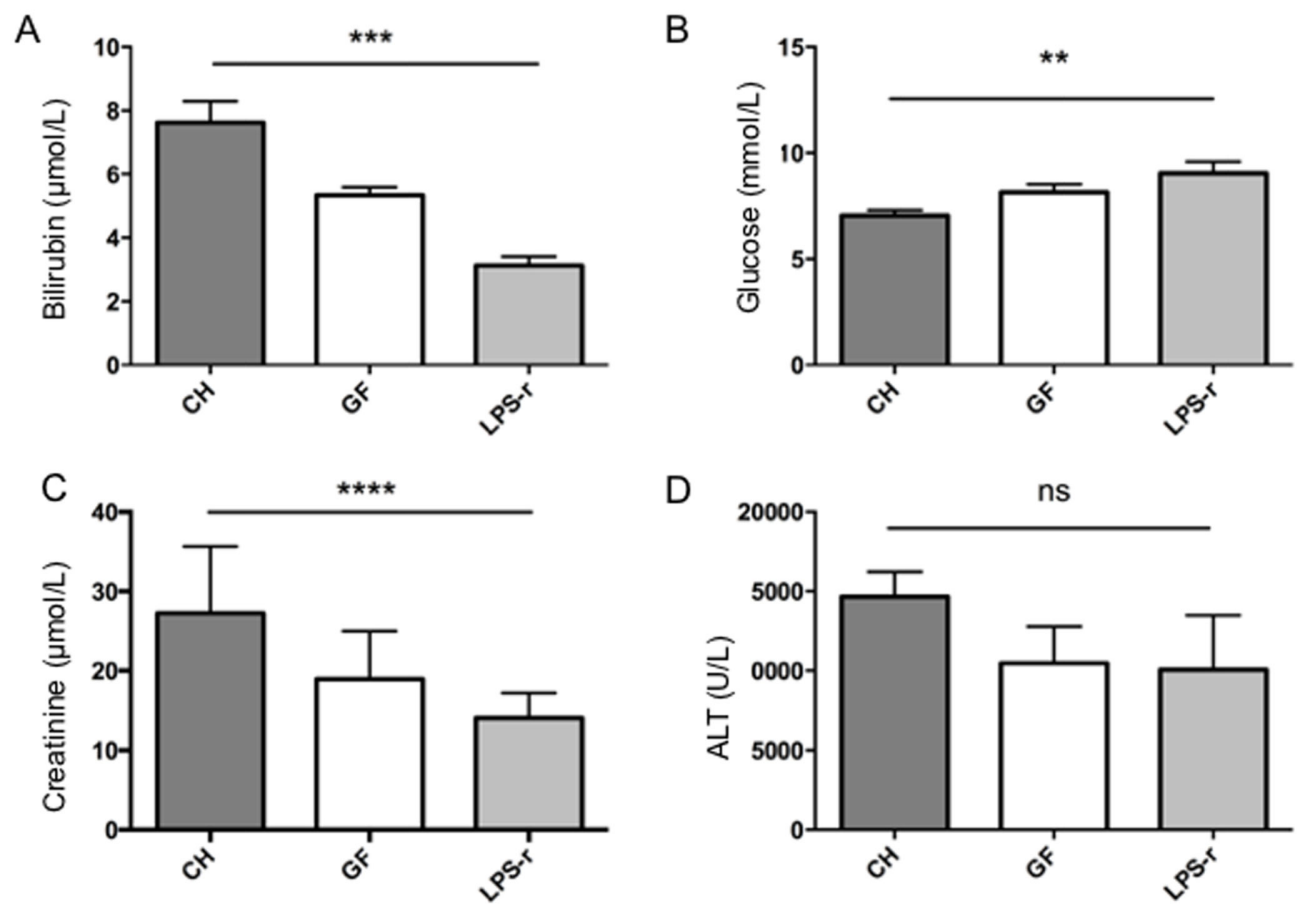
Biochemistry results 8 hours after a toxic acetaminophen dose in experimental groups of germ free (GF, n=9), conventionally housed (CH, n=20) and LPS-resistant (LPS-r, n=10) mice. (A) Bilirubin (p=0.0001) (B) Glucose (p=0.009) (C) Creatinine p=0.0001 (D) ALT p=0.16

At 8 hours post-dosing with acetaminophen, biochemical measures of acute liver failure and multi-organ dysfunction (bilirubin, glucose and creatinine) were assessed in the three groups of mice. There was a significant difference between groups in the bilirubin, glucose and creatinine levels (figure 2, A,B & C). GF and LPS-r mice showed lower bilirubin levels (GF (mean 5.3 +/- 0.3µmol/L), LPS-r (mean 3.1 +/- 0.3µmol/L) than CH mice (mean 7.6 +/- 0.7µmol/L) (Kruskal Wallis *p=0.0001, (Dunn’s CHvGF p=≤0.05, CHvLPSr p=≤0.0001)*) and higher glucose levels (GF 8.14 +/-0.38 mmol/L, LPS-r 9.03 +/-0.57 mmol/L) than CH mice (CH 7.06 +/-0.25 mmol/L) (Kruskal Wallis *p=0.009 (Dunn’s CHvGF p=ns, CHvLPSr p=≤0.01)).* Creatinine levels varied significantly between the three groups with the CH groups having higher levels (27.27 +/-1.9 µmol/L) compared to the GF (18.96 +/- 2.0 µmol/L) and LPS-r (14.05 +/- 1.0 µmol/L) groups (Kruskal Wallis *p=0.0001 (Dunn’s CHvGF p=≤0.05, CHvLPSr p=≤0.0001))*.

ALT values showed considerable variation within each group, but demonstrated good correlation with the hepatic necrosis score (Spearman r=0.78, p=<0.0001). There was no significant difference between the mean ALT for the three groups (CH 14656+/- 6938, GF 10747 +/- 6923, LPS-r 10052 +/- 10834, Kruskal-Wallis *p=0.16*) (see figure 2D).

**Figure 3:**
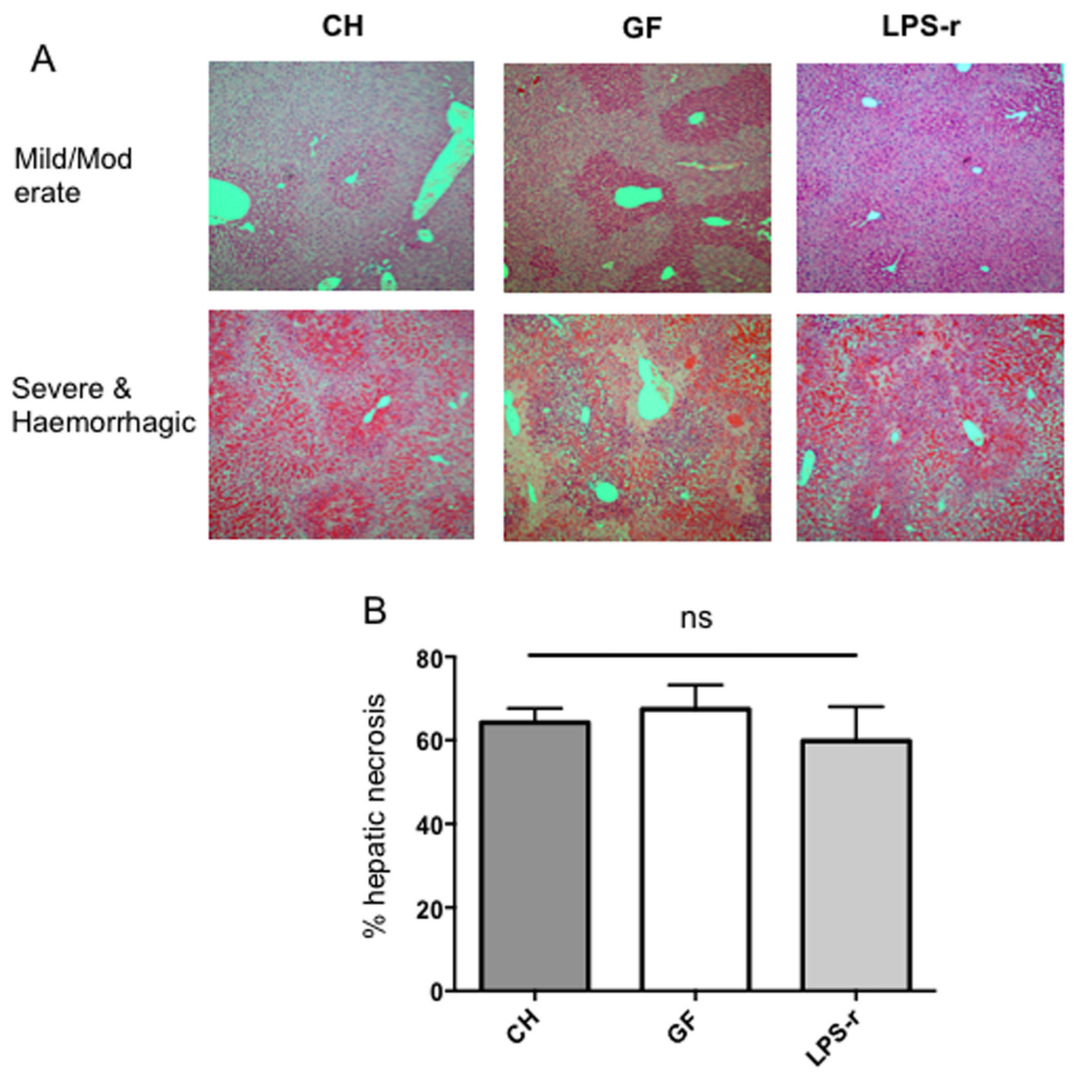
(A) Representative H&E stained sections from each experimental group showing range of hepatic necrosis from mild/moderate to severe and haemorrhagic. Magnification (x 40). (B) Hepatic necrosis scores for each experimental group showing no significant differences between groups.

Review of H&E stained hepatic sections showed the classical appearance expected in acetaminophen toxicity of centrilobular necrosis. In some of the more severely affected mice, extensive hepatic haemorrhage was observed (see Figure 3A for representative images). The mean percentage hepatic necrosis was 64.2% +/- 15.2 (range 37-87%) for CH, 67.4% +/- 16.5 (range 44-84%) for GF and 59.8% +/- 26.0 (range 27-94%) in LPS-r mice. No significant difference was demonstrated between groups (Kruskal Wallis test *p=0.79*) (see figure 3B).

Hepatic cytokines were measured in liver homogenates from the CH, GF and LPSr groups at 8 hours post-injury. There was a non-significant trend for lower levels of NF-κB dependent and inflammasome associated cytokines, TNFα, IL-1β and Il-6 in the LPSr mice (supplementary figure C)

### NMR

#### Baseline urinary spectra & taurine integrals

**Figure 4:**
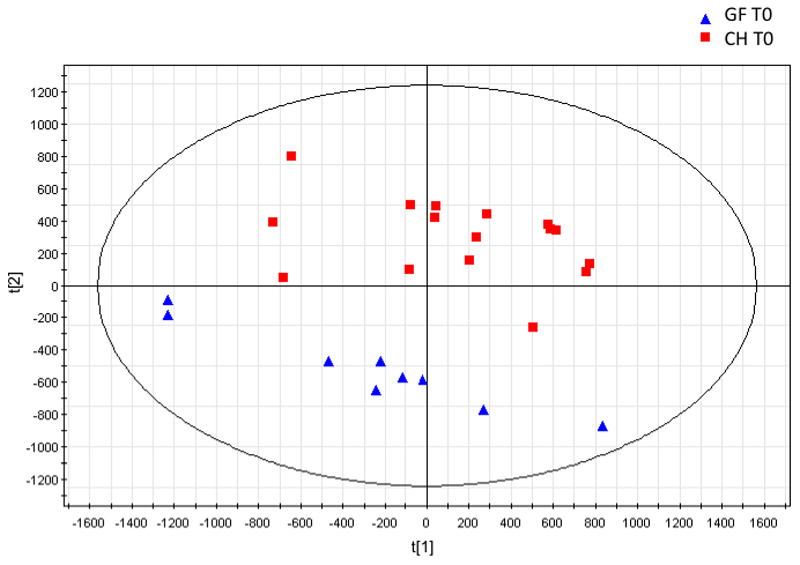
PCA Scores plot of Germ Free (GF) versus Control (C) mice. Two-component model R2(Y)=0.643, Q2(Y)=0.479

Principle components analysis (PCA) and a Partial Least Squares Discriminant Analysis (PLS-DA) of the unfasted (T-24) baseline urinary spectra from GF and CH mice showed a clear separation between groups. Thus suggesting the urinary metabolic profiles of these groups of animals were distinct. On a 2-component PCA model two clusters discriminated along the second PC were noted (R2X=0.625, Q2Y=0.528). A 2-component PLSDA extension gave further discriminatory accuracy (R2Y=0.924, 0.844) with a sensitivity and specificity of 100%. CV-ANOVA statistic was 4 x 10^-8^ and from the permutation analysis the y axis crossing point was 0,24 for R2 and -0.31 for Q2. From this valid model the following metabolites were determined as discriminant from the S-loadings plot: creatine and citrate were increased in GF mice and taurine, TMA and PAG were decreased in GF mice (Figure 4). These findings concur with previously published descriptions of the differences in urinary metabolic profiles between GF and CH mice (18).

Taurine levels were integrated from the urinary ^1-^H-NMR spectra of GF and CH mice at T0. GF mice showed significantly lower baseline urinary taurine than CH control animals (GF 349.7 +/- 125.8 arbitrary units CH 664.9 +/- 165.7 arbitrary units, p=0.0002) (figure 6A)

#### Acetaminophen metabolism

**Figure 5:**
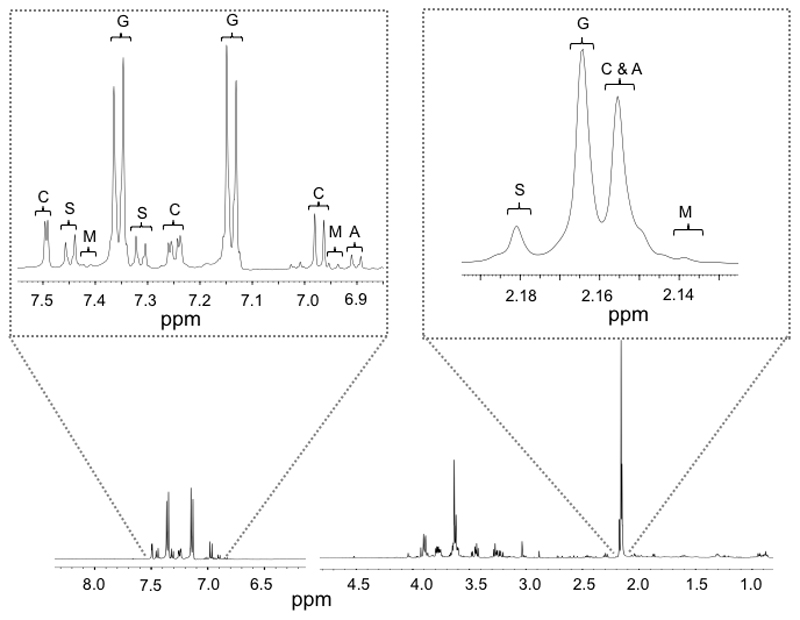
Representative T2 post-dose NMR spectrum demonstrating dominance of acetaminophen-metabolites. Expanded views of the aromatic and aliphatic regions are given illustrating the peak assignments for A, Acetaminophen; S, Acetaminophen-sulphate; G, Acetaminophen-glucuronide; M, Acetaminophen-mercapturate (or *N*-acetyl-L-cysteinyl); C, **L**-cysteinyl Acetaminophen.

**Fig. 6:**
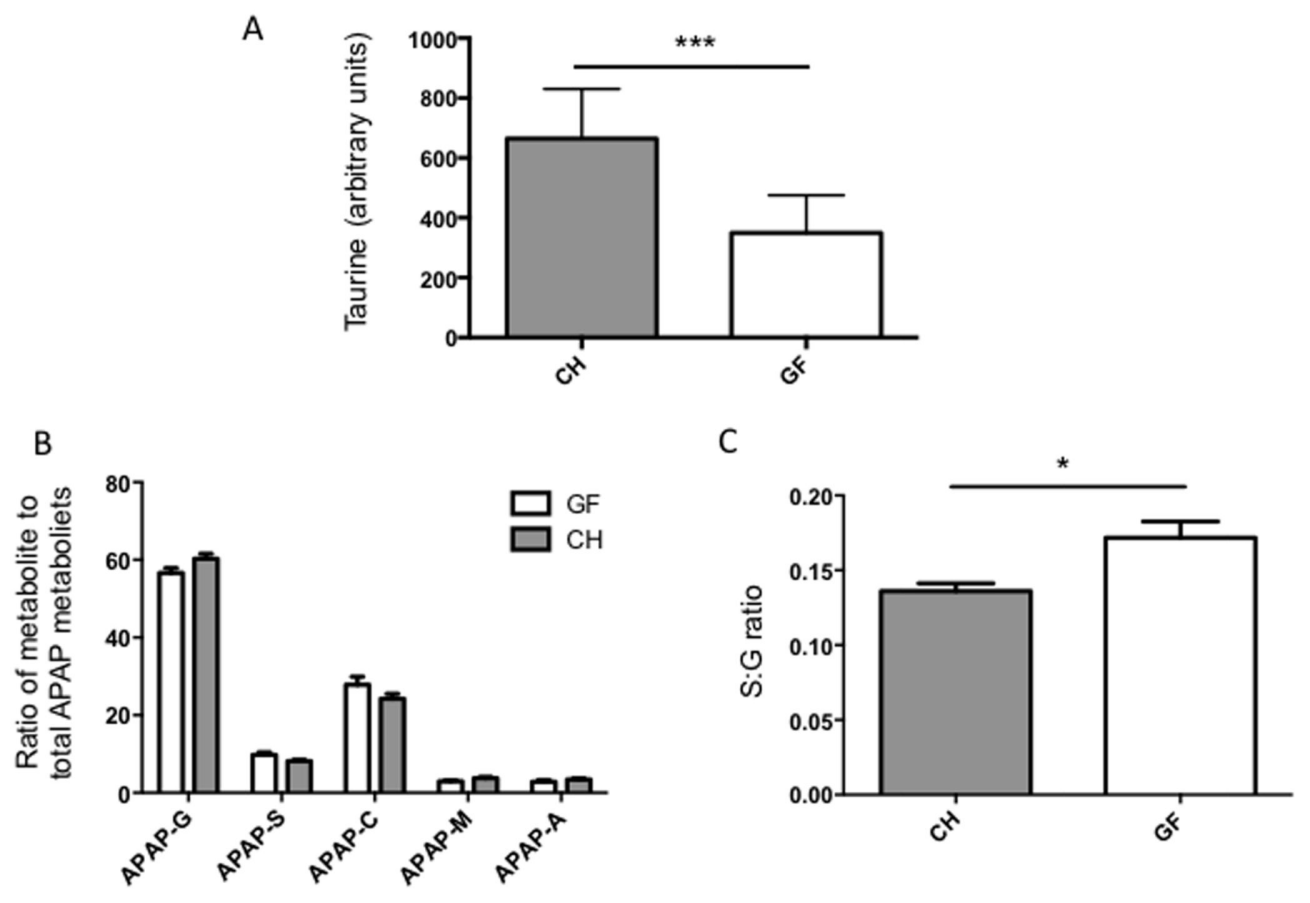
Integrated H^1^-NMR spectra (A) Baseline (T0) urinary taurine levels in GF and CH mice (normalised to total spectral integral) p=0.0002. B and C: Quantification of acetaminophen-related metabolites at 2 hours post dosing expressed as a percentage of total urinary-excreted acetaminophen-related metabolites. (B) Quantification of 5 major urinary acetaminophen-related metabolites in GF and CH mice (C) S:G ratio in GF and CH mice p=0.012.

The metabolic fate of the administered acetaminophen was compared between the CH and GF groups (Figure 5). The urinary acetaminophen-related compounds were similar between the GF and CH mice at T2, with acetaminophen-glucuronide being the dominant metabolite, accounting for 56.6% and 60.3% of the total in GF and CH mice respectively (figure 6B). The S:G ratio, which had been found to correlate with pre-dose urinary gut-derived metabolites in humans, was significantly different between groups at T2 with germ-free mice showing a relatively greater sulphonation capacity (0.136 for CH mice and 0.172 for GF mice (*p=0.012*)) (figure 6C).

#### Metabolic response to acetaminophen hepatotoxicity

**Figure 7:**
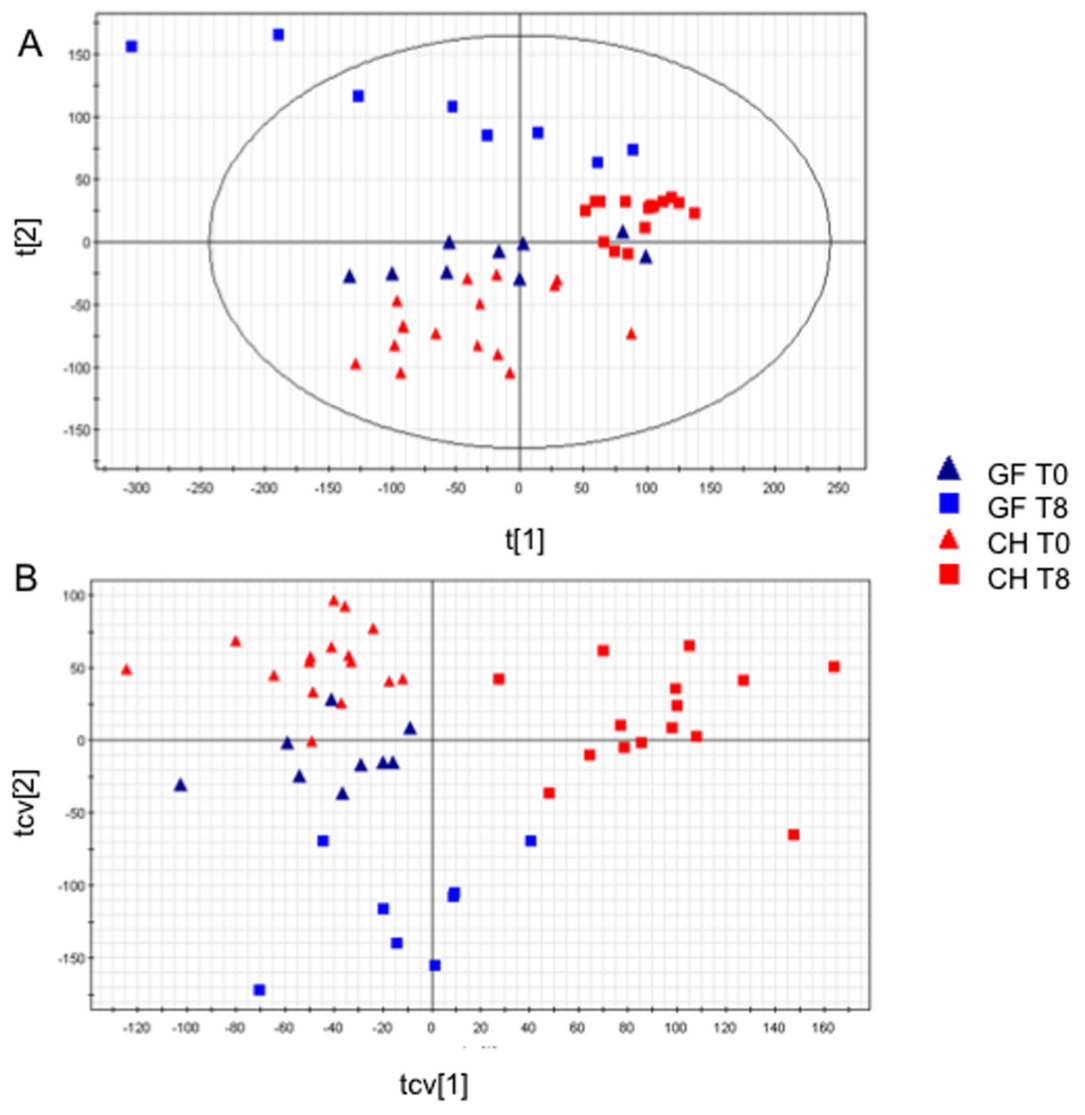
(A) PCA scores plot showing GF and C mice at baseline (T0) and T8. R2(Y)=0.669, Q2(Y)=0.609. (B) Cross validated scores plot from OPLS-DA showing GF and C mice at baseline (T0) and T8. R2(Y)=0.888, Q2(Y)=0.786 using 3 components

Comparing the global urinary NMR response to acetaminophen between the time points T0 and T8 in CH and GF mice at it was clear that the GF mice underwent a more heterogeneous metabolic response. At T8, unsupervised PCA showed distinct clustering in a 2-component model across PC 1 with R2X of 0.77 and Q2 of 0.70. GF status was clearly the discriminating phenotype with GF animals showing a more divergent response from this principal component. The PLS-DA extension had similar robust variance explanation and validation (R2Y=0.93, Q2=0.89, CV-ANOVA p=10^-8^, permutation intercepts R2=0.20, Q2=-0.21, sensitivity, 100% specificity 100% between GF status). The PCA scores plot from a model using all four time points shows this divergence (Figure 7A). Cross validated plots of all 4 time points from an OPLSDA model (R2Y=0.888, Q2Y=0.786) further demonstrate this (Figure 7B). From a model considering the T8 time point only in these 2 groups (R2Y=0.933, Q2Y=0.893) the S loadings plot the residual acetaminophen metabolites were the likely metabolites responsible for this discrimination.

## Discussion

Inter-individual susceptibility to acetaminophen-induced hepatotoxicity has long been recognised by clinicians treating patients who overdose on this medication. One potential source for this variability is the difference in patient’s intestinal microbiota, a factor which has been demonstrated to effect drug metabolism and toxicity in experimental studies. We hypothesised that the absence of intestinal microbiota would attenuate the development and severity of acute acetaminophen-induced hepatotoxicity and that this effect would be partly modulated through reduced LPS-TLR4 signalling. Overall results have shown that in a murine model of acute, severe acetaminophen toxicity the absence of intestinal microbiota is not associated with a greater degree of hepatic injury as shown by hepatic necrosis or ALT levels. However a milder clinical phenotype with less renal impairment, lower bilirubin levels and a trend towards higher plasma glucose was seen in the GF animals.

Interestingly, the inter-individual variability in response to acetaminophen-induced hepatotoxicity was as pronounced in the GF group as the CH group, with similar standard deviations in the mean ALT and hepatic necrosis score. This suggests that differences in intestinal microbiota are unlikely to underlie the frequently observed variability in murine models of acetaminophen toxicity (8).

The slight protective effect of a sterile GI tract was observed despite a minor but significant baseline difference in hepatic glutathione that might pre-dispose germ free animals to enhanced cellular injury by the reactive metabolite of acetaminophen, NAPQI, by limiting detoxification capacity. CYP2E1 mRNA expression and protein levels did not significantly differ between groups at baseline. Our observation that the urinary metabolites of GF and CH mice show no difference in the proportion of drug metabolised via the toxic intermediate supports that CYP2E1 activity is not affected by the presence of intestinal bacteria. Urinary taurine levels have previously been shown to correlate with hepatic taurine and higher levels are associated with protection from hepatotoxicity (19). We have demonstrated that urinary taurine levels are significantly lower in GF mice than conventional controls, a factor which again might be expected to predispose GF mice to a more severe response to acetaminophen. Overall these findings imply that the protective effects of GF status impact at a later stage in the development of hepatotoxicity, after the initial metabolism of acetaminophen.

Analysis of urinary acetaminophen metabolites demonstrated that drug metabolism is affected by the presence of intestinal microbiota, with germ free animals displaying a lower urinary ratio of glucuronated:sulphonated acetaminophen 2 hours after dosing. This shift in metabolism may be due to competitive inhibition of hepatic sulphonation capacity by microbial-derived aromatic compounds in those animals with bacterially colonised intestines. Early work using gradient elution HPLC to quantify acetaminophen metabolites in the urine of germ free mice also described this relationship, with a significant increase in the percentage excretion of acetaminophen as a sulphonated conjugate in germ free animals (20). This observation is also consistent with the human study by Clayton et al which showed a decrease in the S:G urinary ratio in healthy subjects with higher pre-dose bacterially derived p-cresol sulphate levels (10). In our study, the total quantity of acetaminophen-sulphate and glucuronide in the urine did not differ between the CH and GF groups, suggesting any reduced sulphonation capacity in CH mice was at least partly compensated for by enhanced glucuronation and thus subsequent toxicity is unlikely to have been affected by this alteration.

The main differences in the biochemical markers of liver impairment between the GF and CH groups were protective effects on the development of hyperbilirubinaemia, renal impairment and hypoglycaemia in GF mice. Of interest is the observation that the LPS-r group of mice, with a genetic polymorphism in their TLR4 receptor, have an even greater protection from renal impairment, hyperbilirubinaemia and hypoglycaemia, compared to GF mice. There is also a non-significant trend for lower levels of cytokines associated with TLR4 and inflammasome activation in these mice. Overall, this suggests that these mice have an additional protective factor, beyond simply resistance to LPS. A possible explanation for this observation is that the TLR4 receptor that is mutated in this mouse strain functions not only as a PAMP (pathogen-associated molecular pattern) receptor, identifying the bacterial product LPS, but also recognises HMGB1 an important DAMP (damage-associated molecular pattern) in sterile inflammatory conditions (21). HMGB1 has been shown to have an important role in acetaminophen-induced ALF, being a prognositic marker in human subjects and in murine models (22, 23). Thus the LPS-r mouse, with its global impairment in TLR4 signalling, that affects the recognition of HMGB1 and LPS, has a milder response to liver injury. TLR4 knockout mice have been shown to be relatively protected from acute sterile liver injury and the development of extra-hepatic organ dysfunction after liver injury (24)(25)(26). Studies have also demonstrated that TLR4 antogonists may reduce the extent of liver injury in acetaminophen and galactosamine induced liver failure models (24, 27).

This study has a number of limitations. The use of a high dose of acetaminophen to model acute, severe injury necessarily limited the study duration due to animal welfare considerations. In this respect we have only modelled and investigated the earlier phases of acetaminophen-induced acute liver injury: when toxicity, hepatocellular necrosis and organ failure first develop. The later stages, seen in human patients who go on to develop established ALF with secondary immune dysfunction and multiple-organ failure, were not represented. Intestinal microbiota may be particularly influential at this later stage as evidenced by the divergent metabolic responses of the GF and CH groups and developing organ dysfunction in the CH group. In our murine model the route of delivery of acetaminophen was IP as it is preferable to oral gavage in terms of welfare and dosing consistency. However this differs from patients in whom acetaminophen overdose is almost always taken orally. When administered orally, acetaminophen is rapidly absorbed from the proximal small intestine with minimal metabolism by the intestinal wall, hence in both IP and oral dosing the majority of drug is bioavailable to the liver during first pass metabolism (28, 29). The effects of intestinal microbiota are likely to be either remote, in the case of LPS stimulation or during the entero-hepatic circulation of acetaminophen metabolites and therefore will not be affected by the initial drug delivery method

In conclusion, we have demonstrated that in an acute toxicity model the presence of intestinal microbiota influences the metabolism of acetaminophen by reducing hepatic sulphonation capacity. Germ free mice experience a milder hepatotoxicity phenotype with lower plasma bilirubin, lower creatinine levels and a trend towards higher glucose compared to conventionally housed animals, despite no significant differences in the extent of their liver lesion as defined by histology and plasma ALT. Impairments in TLR4 signalling were associated with an even greater protection than GF status alone, suggesting DAMP signalling through TLR4 is plays a significant role in the pathogenesis of acetaminophen toxicity. Overall this study suggests that the protective effects of a sterile intestine impacts late in the evolution of acute liver failure, on the metabolic response to initial injury and the development of extra-hepatic dysfunction such as renal impairment. In this respect the study findings are of particular clinical relevance as delayed patient presentation means the opportunity for intervention in acetaminophen-induced acute liver failure is often after the initial liver injury is established. This study therefore raises the possibility of therapeutic intervention to modify intestinal microbiota through gut cleansing antibiotics or pharmacological inhibition of TLR4 signalling in the treatment of patients with acute liver failure.

## Supplementary Material

Supplementary Figures
