# Supplementary material for: The role of intestinal microbiota in murine models of acetaminophen-induced hepatotoxicity
Source: Liver Int. Author manuscript; Available in PMC 2018 Mar 28. (PMC5873516; doi:10.1111/liv.12689)

**The role of intestinal microbiota in murine models of acetaminophen-induced hepatotoxicity**

**Lucia A Possamai^1^, Mark JW McPhail^1^, Wafa Khamri^1^, Bishan Wu^2^, Danilo Concas^2^, Mark Harrison^2^, Roger Williams^3^, Roger D Cox^2^, I Jane Cox^3^, Quentin M Anstee^4^, Mark R Thursz^1^**

^1^ Department of Hepatology, Imperial College London W2 1NY, UK

^2^ MRC Mammalian Genetics Unit, Harwell, Oxford, OX11 0RD, UK

^3^ Institute of Hepatology, Foundation for Liver Research, 69-75 Chenies Mews, London WC1E 6HX, UK

^4^ Institute of Cellular Medicine, Newcastle University, Newcastle upon Tyne, NE2 4HH, UK

**Published:** Liver Int. 2015 Mar;35(3):764-73. doi: 10.1111/liv.12689. Epub 2014 Oct 8. PMID:25244648 Open Access, Creative Commons licence

**Corresponding Author:** Lucia A Possamai. Liver & Antiviral Unit, QEQM Building, St Mary’s Hospital, Praed Street, London W2 1NY, UK. [l.possamai@imperial.ac.uk](mailto:l.possamai@imperial.ac.uk)

Telephone: (0044) +2033126454 Fax: (0044)+2077249369

**Supplementary Figure S1**

Cytokine levels measured in hepatic homogenates 8 hours after acetaminophen administration. No significant differences by one way ANOVA with multiple comparisons test. (samples run in duplicate, each bar represents *n* = 8 mice).


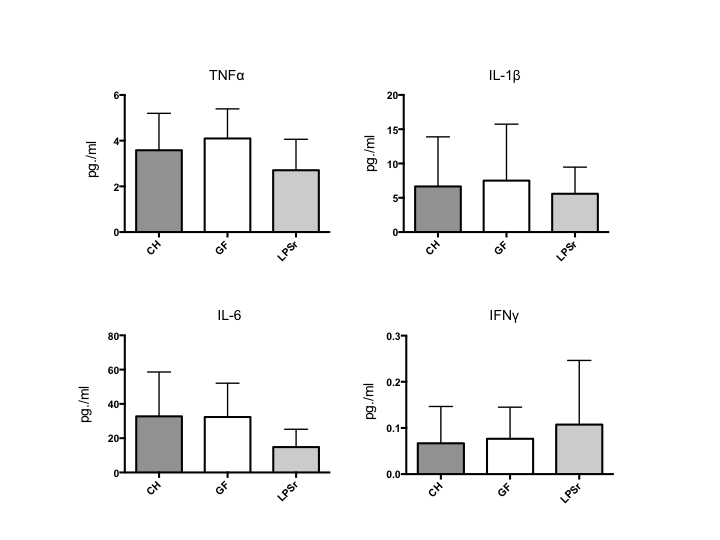


**Supplementary Figure S1a**


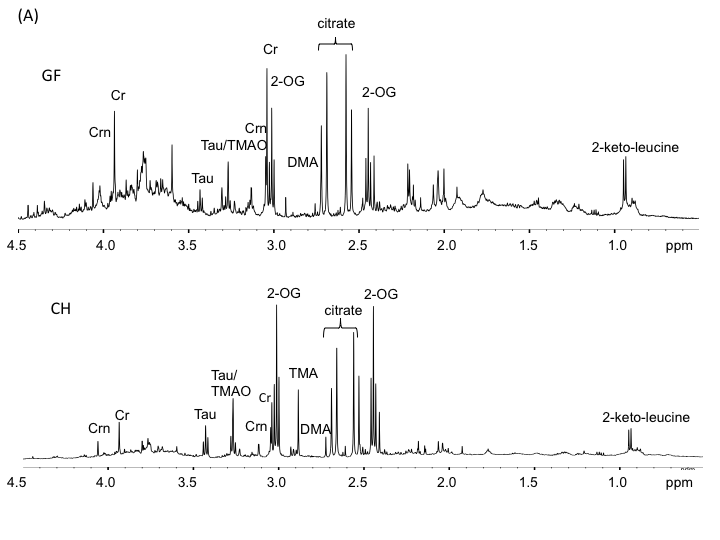


**Supplementary Figure S1b**


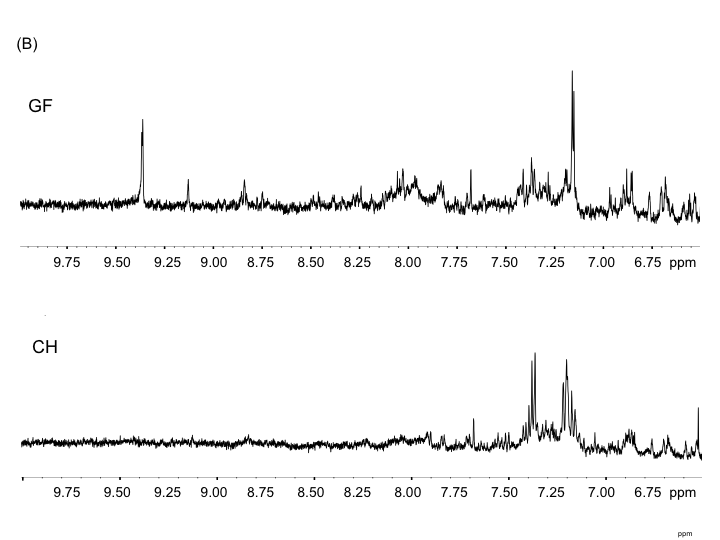

Supplement: Supplementary Figures [file NIHMS76722-supplement-Supplementary_Figures.docx]
